# The Effects of Proportional Processing and Multiple Opponents on Contest Assessment in Male Green Swordtail Fish (*Xiphophorus hellerii*)

**DOI:** 10.1093/icb/icaf084

**Published:** 2025-09-16

**Authors:** P A Green, L A Kelley, E M Caves

**Affiliations:** Department of Ecology, Evolution and Organismal Biology, Brown University, Box G-W, 80 Waterman St., Providence, RI 02906, USA; Department of Ecology, Evolution and Marine Biology, University of California, Santa Barbara, CA 93106, USA; Centre for Ecology and Conservation, University of Exeter, Penryn TR10 9FE, UK; Centre for Ecology and Conservation, University of Exeter, Penryn TR10 9FE, UK; Department of Ecology, Evolution and Organismal Biology, Brown University, Box G-W, 80 Waterman St., Providence, RI 02906, USA; Department of Ecology, Evolution and Marine Biology, University of California, Santa Barbara, CA 93106, USA; Centre for Ecology and Conservation, University of Exeter, Penryn TR10 9FE, UK

## Abstract

When animals compete over essential and limited resources, how they gather information about fighting ability is a crucial factor influencing their decision-making. Most research in animal contests asks how decisions are made when facing a single competitor; however, in many cases, individuals face multiple potential opponents and may incorporate information on this social environment. In addition, recent research suggests that animals perceive contest-relevant stimuli like body size in a proportional, not absolute, manner; this proportional processing has rarely, if ever, been incorporated into studies of contest assessment. Green swordtail fish (*Xiphophorus hellerii*) live in social aggregations, in which males may defend females from multiple potential opponents. Here, we asked how focal male green swordtails defended live females when presented with two simulated males that differed by known sizes. We found that focal males spent less time near the larger, more salient, of the two competitors as the mean size of both simulated competitors increased. That is, focal males mainly used information on the social environment to make competitive decisions, as opposed to information about own or relative fighting ability as commonly assumed in most contest theory. We also found that males who spent less time with the largest competitor shifted their attention to the defended female, devoting more time near this resource. Our findings suggest that, when there are multiple potential competitors, common models of decision-making in contests may be less applicable than previously assumed. Further, given the common use of proportional processing across animals, we suggest that future work on contests incorporates this type of perception.

## Introduction

Across diverse animal taxa, contests dictate access to resources that are essential to survival and reproduction, such as food, shelter, and mates (Briffa and Hardy 2013). Because of their impact on fitness, contests exert strong selective pressure on animal traits, including morphology and behavior (Andersson 1994; Searcy and Nowicki 2005; Emlen 2008). In particular, the behaviors that competitors use to gather information about themselves and/or their opponents—and how this information influences decisions like when to give up the fight—has long been a central focus of research in animal behavior (Arnott and Elwood 2009).

These information-gathering strategies, or “assessment strategies,” were first derived from evolutionary game theoretical models over 50 years ago (Maynard Smith and Price 1973; Maynard Smith 1974). Most often, theory and experiments in assessment focus on two individuals engaged in a contest over a single resource, such that each competitor may assess its own fighting ability (termed resource holding potential, or RHP), its opponent’s RHP, and/or the value of the contested resource (Arnott and Elwood 2009; Sherratt and Mesterton-Gibbons 2013). For assessment of RHP in particular, two main strategies dominate the literature. In a “self-assessment” strategy, a competitor does not gather information about its opponent’s RHP; instead, it simply persists in the conflict until it reaches a self-imposed threshold of fighting costs. By contrast, in “mutual assessment,” a competitor gathers information about opponent RHP (e.g., via signaling) and uses this information, along with information about its own ability, to make a decision about when to give up the contest (Arnott and Elwood 2009; Green and Patek 2018). Resource value assessment, by informing how motivated competitors are to fight, can further modify fighting behaviors. For example, in systems where males compete for access to female mates, male competitors may place higher value on more fecund females, and therefore fight harder to secure mate access (Arnott and Elwood 2008).

While most work in assessment strategies takes this two-competitor approach, in many contests, other nearby conspecifics may affect the decisions competitors make, either before or during a contest (Sherratt and Mesterton-Gibbons 2013). For instance, individuals may change their future fighting behavior after viewing contests between other competitors (Oliveira et al. 1998; Hotta et al. 2015). In addition, individuals near two competitors—termed “third parties”—may influence fighting behavior, or even participate in the contest themselves (Jennings et al. 2009).

In addition to the added complexity that comes with accounting for nearby conspecifics, recent work has highlighted the importance of incorporating sensory perception into our understanding of assessment. Historically, empirical studies and theoretical models of animal signals—including assessment signals used during contests—have assumed that individuals perceive continuous signal variation (e.g., size, color, amplitude) in a linear fashion (Peniston et al. 2020). By contrast, recent work is revealing that many animal taxa perceive this continuous variation in non-linear ways (Caves et al. 2019; Green et al. 2020; Caves and Kelley 2023). In particular, magnitude-based traits like the size of an individual’s body or body part (e.g., a weapon)—traits that are often main proxies for RHP—are often perceived using proportional processing (Akre and Johnsen 2014; Bullough et al. 2023). Proportional processing adheres to Weber’s Law, meaning that individuals use proportional size differences rather than absolute size differences in discriminating between stimuli of different magnitudes.

**Fig. 1:**
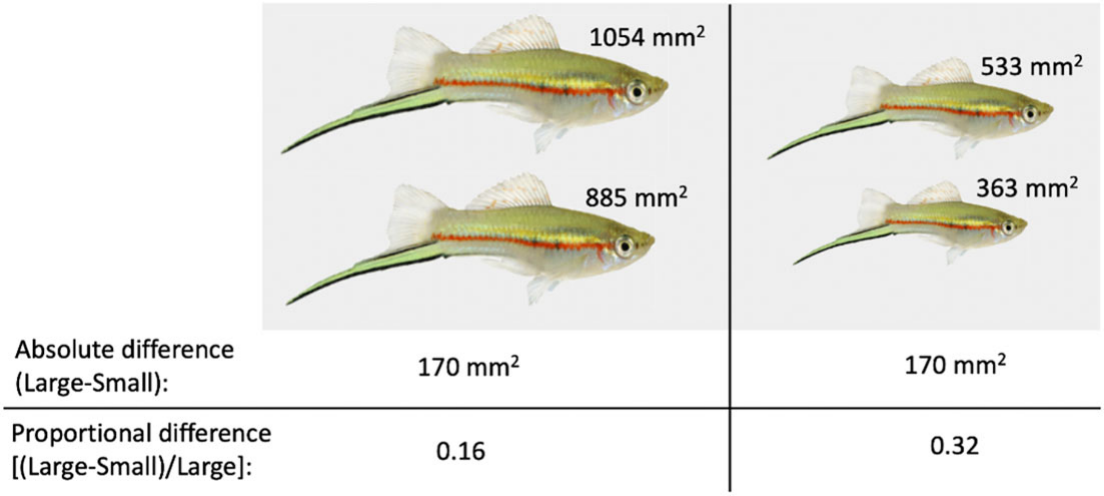
Proportional processing. Each pair of fish (left side and right side) differ in lateral surface area by the same absolute amount, 170 mm^2^. However, because the fish on the right side are smaller, the proportional difference between the two individuals in the right pair is greater than the difference between the two in the left pair. Thus, it is easier to tell which is the larger individual in the right pair than it is in the left pair.

Consider two pairs of stimuli that differ from one another by the same absolute amount. Depending on whether both stimuli in each pair are generally large or small, the proportional magnitude difference between the stimuli within a pair can differ greatly between pairs, even though the absolute difference is the same (Fig. 1). For instance, if competitors differ in absolute body size by 2 mm, the proportional difference (difference in size divided by size of the larger stimulus) between them is much greater if the competitors are smaller (e.g., 3 mm v. 5 mm, proportional difference = 0.4) than it is if the competitors are larger (e.g., 30 mm v. 32 mm, proportional difference = 0.06, Fig. 1). Thus, under proportional processing, the ability to discriminate between two stimuli depends not only on the difference between them, but also whether the two stimuli are small or large (Caves and Kelley 2023), as larger proportional differences (i.e., those between smaller stimuli) are easier to detect. Proportional processing is well-known in humans, and has been demonstrated in perception of signals in mate choice in non-human animals (Akre et al. 2011; Caves and Kelley 2023). However, proportional processing has rarely, if ever, been investigated in the context of competition. Understanding how proportional processing affects contest decisions, including in scenarios like the presence of other conspecifics, can add more realism to our understanding of animal contests. In turn, this may inform future experimental approaches and theoretical models, as well as our understanding of how contests affect evolution.

Here, we test how the presence of third-party individuals and the use of proportional processing affects the decisions individuals make during contests over mate access. Male green swordtail fish (*Xiphophorus hellerii*) compete over access to females as mating resources (Magellan and Kaiser 2010). While males are known for their elongated, sword-like tails, overall body size is a proxy for RHP, such that larger males tend to win fights (Prenter et al. 2008) (see also (Moretz 2003) for *X. cortezi*). Prior work has shown that the presence of nearby individuals affects fighting decisions in swordtails. For instance, previewing other competitors engaging in a contest affects an individual’s future fighting behavior (Earley and Dugatkin 2002), as does viewing a future opponent in a non-competitive context (Earley et al. 2003). Our goal was to add to this background by asking how proportional processing of body size affects fighting decisions, and how the presence of a conspecific *during* a contest affects assessment and decision-making.

We used a two-choice paradigm and video playback of animated rivals to test how a single male associated with simulated competitors when in the presence of a defensible resource (a live female). We measured the time that the focal male spent associating with the largest simulated rival, which we presumed the focal male would perceive as the most salient competitor. Although one prior study found support for mutual assessment during dyadic contests in this species (Wilson et al. 2011), no work (to our knowledge) has tested assessment among multiple competitors; thus, we did not have a directional hypothesis of what assessment strategy we expected competitors to use. Rather, using a model comparison approach, we identified the assessment strategy that best described focal male behavior. In addition, we asked whether and how males altered the time they spent with the smaller of the stimulus males, and with the defended female, as a result of their assessment of the larger stimulus male.

## Methods

### Methods overview

In brief, we measured the amount of time that a focal male associated with a live female (a resource) and animations of two simulated rivals that differed in lateral projection area [hereafter “size,” which we equate with RHP (Prenter et al. 2008)] from both the focal male and from each other. We used model comparison approaches to test whether the focal male’s association with the larger competitor, which we assumed was the most salient competitor, was consistent with self-assessment (focal male size only) or mutual assessment (focal male size relative to largest competitor size). We also tested whether association was consistent with assessing both competitors in relation to each other, which we termed “social assessment.” Specifically, we examined two types of social assessment, which tested whether males assessed (1) the size difference between both competitors, or (2) the mean size of the two competitors. In our models that involved size differences, we used the proportional size difference, because female *X. hellerii* have been shown to proportionally process body size (Caves and Kelley 2023).

### Animal care and ethics

Animals in this experiment were treated in accordance with the ethical guidelines of the University of Exeter (ethics approval eCORN002243). Fish handling and experiments were carried out by P Prentice (Home Office Personal License I2099DA1E) and S Green (I54451846), under Home Office Project License PF6E68517. Fish used in this experiment were sexually-mature descendants of a wild-derived population collected in Belize in 2002. Males and females used in this experiment were housed in mixed-sex groups, apart from 11 males that were kept in single sex groups for husbandry purposes. Fish were fed a mixture of bloodworm, mysis shrimp, and artemia each morning and flake food (ZM Flake, Fish Food and Equipment, Hampshire UK) each evening. Water temperature was kept between 22 and 24°C and tanks were lit from above with AquaBeam LED lights (Tropical Marine Centre Ltd, Herefordshire, UK) on a 12:12 light:dark cycle. Any fish that could not be uniquely identified by their size and sword color were tagged subcutaneously under anaesthesia with an individually-identifiable combination of colored elastomer tags by L Kelley (Home Office Personal License I92638227), prior to the start of data collection (Northwest Marine Technology Inc, Washington, USA).

### Behavioral experiments

We used a dichotomous-choice behavioral paradigm to determine how males, in the presence of a female, responded to 2-D animations of courting males that were identical to one another aside from differing in size. Two-dimensional animations of courting males are a widely used tool in fish (Baldauf et al. 2009; Fischer et al. 2014; Levy et al. 2014), including in swordtails (Morris et al. 2003) and in our specific swordtail population (Caves and Kelley 2023, 2025), for examining how fish vary their behavior toward other individuals with experimentally-manipulated traits (e.g., body size). Although the use of live rival males, instead of animated males, may have added more “social realism” to our experiments, the use of animated males allowed us to precisely control the body size of each rival while ensuring each “behaves” in a similar way. This control and consistency is critical when testing proportional processing, because it isolates the effect of (proportional) size on focal male competitive behavior.

### Stimulus design

**Fig. 2:**
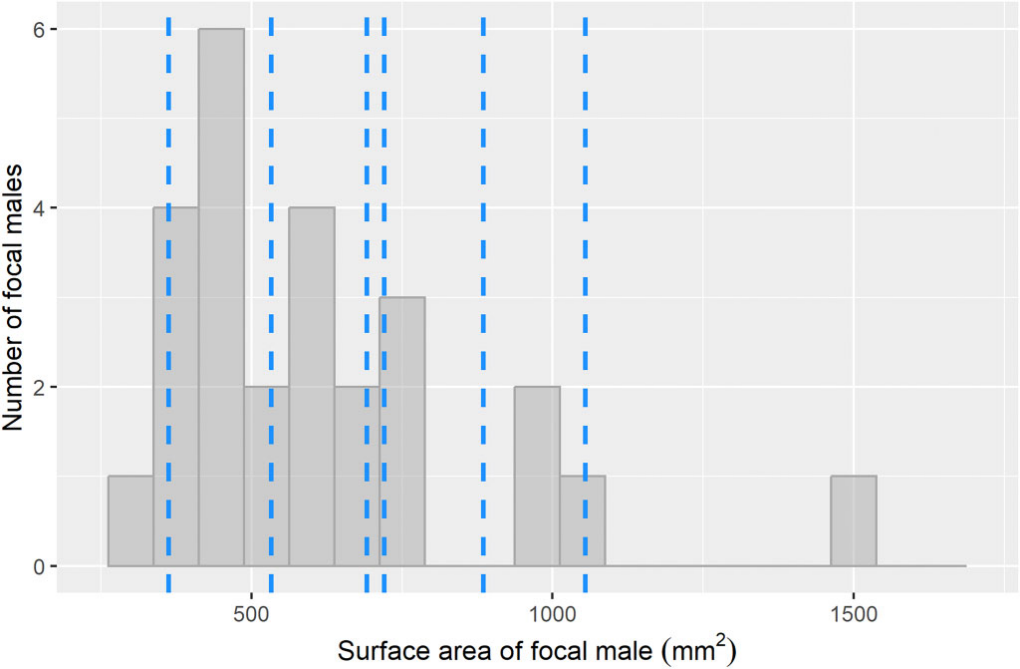
Distribution of focal males and stimuli. The distribution of focal male size (*x*-axis, gray bars and density plot) was not different from the size distribution of individual simulated rival males (vertical dotted lines).

To create stimuli, we took a photograph of a male swordtail in our experimental population whose body (44 mm) and sword length (32 mm) were within one standard deviation of the population mean (mean ± standard deviation body size: 42.5 ± 5.31 mm; sword length: 28.5 ± 9.97 mm). In the photograph, the fish was separated from the background using Adobe Illustrator, and the size of the fish was calibrated such that the size of the stimulus when displayed on the tablets used in trials (Samsung Galaxy Tab 10.1, Samsung Corp; 22.3 cm × 14 cm screen, 1200 × 1920 pixel resolution, 60 Hz refresh rate) equaled the size of the fish in real life. This original image was then scaled to create male stimuli of larger and smaller lateral areas, following (MacLaren and Daniska 2008). These stimuli spanned most of the range of focal male body size, and the distribution of stimulus body size was not different from the distribution of focal male body size (Wilcox test W = 98, *P* = 0.36, Fig. 2).

We then used Microsoft PowerPoint (version 16.57) to animate the stimuli. A male image was first superimposed onto a blank, bright grey (RGB: 238, 238, 238) background (following Baldauf et al. 2009), and animations were created using PowerPoint “animation paths.” Specifically, animations were included to represent males (1) swimming from one side of the tank to the other, moving off the screen, and reappearing swimming in the other direction over the course of 30 s (following (Baldauf et al. 2009; Fischer et al. 2014; Thünken et al. 2014); and (2) performing backward-swim maneuvers, a courting and low-level aggressive behavior performed by male green swordtails, at a rate of 3 backward-swim maneuvers every 60 s (Rosenthal et al. 1996; Royle et al. 2005). All aspects of the animations—stimulus and background color, location of stimuli on the screen, and path and speed of stimulus—were identical across stimulus males, with only the size of the male varying.

Overall, we used six animated stimuli in our experiments, presented in seven pairs. Pairs were created to meet two criteria. First, all size differences were theoretically resolvable, given the visual acuity of male green swordtails (∼2 cycle per degree (Caves et al. 2021)) and the size of our experimental tanks. Second, pairs covered a broad range of both absolute (169 mm^2^ to 691 mm^2^) and proportional (range: 0.16 to 0.66) differences in body size (lateral surface area), with absolute difference in body size calculated as:


\begin{eqnarray*}
{{{\mathrm{A}}}_{\mathrm{l}}}{\mathrm{- As}}
\end{eqnarray*}

and proportional difference calculated as:


\begin{eqnarray*}
\left( {{{A}_l}-As} \right)/{{A}_l}
\end{eqnarray*}

where *A_l_*is the area of the larger stimulus and *A_s_* is the area of the smaller stimulus.

### Two-choice trials

**Fig. 3:**
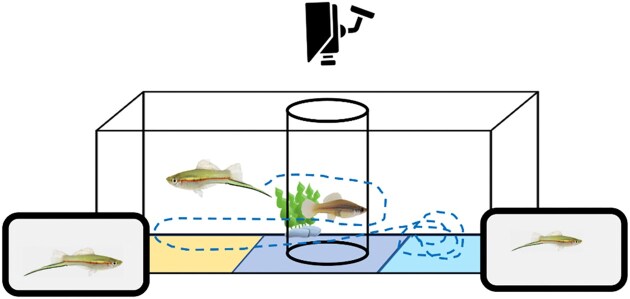
Two-choice trial methods. The focal male (fish in large, central tank) defended a female (inside cylinder in middle) from two animated stimulus males presented on tablets on either side of the tank. Focal male movement (dashed line) was filmed from a camera above the tank (camera icon), and the time the focal male spent in each of three virtual zones, i.e., associating with each stimulus male (larger = yellow area on left-hand third of tank, smaller = light blue area on right-hand third of tank) and near the female (dark blue area in central third of tank), was calculated.

Two-choice trial methods are visualized in Fig. 3. Trials were conducted in a two-choice tank (45.7 × 25.4 × 25.4 cm) filled to a depth of 15 cm using water from the home tank system. All trials were filmed from above using a camera (Sunkwang C160 video camera, 6–60 mm manual focus lens) suspended above the tank. The camera was connected to a computer running the Viewer tracking software (BiObserve), which virtually divided the tank into three equally sized zones and tracked all movements made by the fish for the duration of a trial. To improve the accuracy of the automated tracking, the experimental tank was lit from underneath (following e.g., Houslay et al. 2018; Prentice et al. 2020) with a lightpad (UltraSlim LED LightPad, MiniSun Limited, Manchester, UK). To prevent external visual disturbance during the trial, a cardboard screen was placed around the tank.

Before the start of a trial, a focal male and a randomly-selected female (i.e., the resource, mean ± standard deviation of standard length 47.4 ± 3.5 mm) were each placed inside of separate clear acrylic cylinders (male 15 cm diameter, female 10 cm diameter) in the center zone of the tank for a 15 min acclimation period. Throughout this acclimation period, tablets that displayed only a plain gray background were placed against each end of the tank. Following the acclimation period, trials began. During a trial, each tablet would first display one minute of plain gray followed by one minute of a male stimulus. During this time, the male was still constrained to the cylinder, to ensure that he viewed both male stimuli from the center, and thus from a consistent distance. We then removed the cylinder from around the male (but not the female, who remained constrained to the center of the tank for the entire trial in order to control for male interactions with the female), to allow the male to access the entire tank while male stimuli continued to play at each end of the tank for three further minutes.

Following trials, males and females were returned to their home tanks, and Viewer extracted the amount of time (in seconds) that the male had spent in each zone, i.e., spent with each male stimulus (end zones) or the female (center zone). After 48 h, the same procedure was followed with the same focal male and female and the same pair of male stimuli; during the second trial, however, each stimulus was presented on the opposite side of the tank to account for possible side biases. Trials were run concurrently with two experimental tanks, so for a given set of stimulus pairs all focal males experienced one of two females. Two new females were used for each set of stimulus pairs. All males were shown the stimulus pairs in the same order. Presentation order had no effect on how much time a male spent with the larger male (linear mixed model with random effect of fish identity, presentation number β ± SE = −1.66 ± 2.22, χ^2^ = 0.56, *P* = 0.46), smaller male (β ± SE = 1.15 ± 1.97, χ^2^ = 0.34, *P* = 0.56), or female (β ± SE = 0.77 ± 2.53, χ^2^ = 0.09, *P* = 0.76).

In total, 26 males were presented with seven stimulus pairs, with two trials per stimulus pair (over two 3-min periods). If a male exhibited stress symptoms (rapid darting back and forth across the tank) during a trial, that trial was rerun at a later date; if stress symptoms were still exhibited on the second trial, that male was excluded from further analysis for that comparison. Out of a total of 399 trials, excluding trials where males showed stress resulted in 330 trials for analysis.

### Statistical analyses

All statistical analyses were conducted in R Version 4.2.1 (R Core Team 2020).

From the raw data from each trial, we calculated the time each male spent associating with the largest male across the two, 3-min-long trials for each stimulus pair (6 min total). We called this variable “association time.” We built seven Linear Mixed Models [LMMs, lme4 package (Bates et al. 2015)] testing how association time was predicted by predictor variables relevant to assessment strategies. These models are also listed in Table 1; the predictors in these models include the size of the focal male (“focal male size”), the mean size of both stimulus males (“mean stimulus size”), the proportional difference in size between the two stimulus males (“proportional stimulus difference”), and proportional difference in size between the focal male and the largest stimulus male (“proportional difference from largest”). All models also included a random effect of focal fish identity, to account for repeated trials on each focal male. We also created a “null model,” in which association time was only predicted by a model intercept and the random effect of focal fish identity. We checked for collinearity between predictor terms in each model using Variance Inflation Factor (VIF) scores (Zuur et al. 2010), with the car package (Fox and Weisberg 2019).

We compared the fit of all seven models using AICc scores, as executed by the model.sel function in the MuMIn package (Barton 2019). This function also calculates the model weight for each model, as:


\begin{eqnarray*}
\textit{model}\ \textit{weight} = \ \frac{{{{e}^{ - \frac{1}{2}\Delta \textit{AICc}}}}}{{\mathop \sum \nolimits_n^1 {{e}^{ - \frac{1}{2}\Delta \textit{AICc}}}}}
\end{eqnarray*}

where *n* is the total number of models in the model set and and ΔAICc is the difference in AICc score between the model and the best-fit model. We used the table of model AICc information (Table 1) to understand which model fit the data best and, therefore, which assessment strategy best described focal male behavior.

**Table 1:** Summary of model comparison results for time spent with the largest stimulus male

Model name	Model predictors	AICc	ΔAICc	Weight
Social mean	Mean stimulus size	1813.8	0.00	0.57
Mutual assessment + social mean	Proportional difference from largest + mean stimulus size	1816.1	2.32	0.18
Null	N/A	1817.3	3.52	0.10
Social proportional	Proportional stimulus difference	1818.4	4.57	0.06
Mutual assessment	Proportional difference from largest	1819.2	5.43	0.04
Self-assessment	Focal male size	1819.4	5.61	0.04
Mutual assessment + social proportional	Proportional difference from largest + proportional stimulus difference	1820.7	6.87	0.02

All models included a random effect of fish ID to account for repeated observations (see the section “Methods”).

Our main analysis used the proportional difference in size between stimulus males, or between the focal male and stimulus males, as predictor variables. This is because prior work has shown that female green swordtails use proportional processing when assessing male size (Caves and Kelley 2023). We also we built the same series of models as described above, but used absolute size difference, instead of proportional size difference. The AICc table ranking these models is presented in Supplementary Table S1; results are similar across the two analyses.

Once we identified the best-fit model that predicted focal male association time with the largest male, we then asked how the predictor(s) from this model also predicted association time with the smaller stimulus male and with the female (which we also calculated from the raw data). We built the same model formula as the best-fit model, but changed the response variable to either association time with the smaller male, or association time with the female. In a *post-hoc* analysis, we also used model comparison to test how the predictors used in the seven models used to analyze time with the largest male (described above) described variation in the time spent with the smaller male and, separately, the female (see the section “Results,” and Supplementary Tables S2 and S3).

## Results

**Fig. 4:**
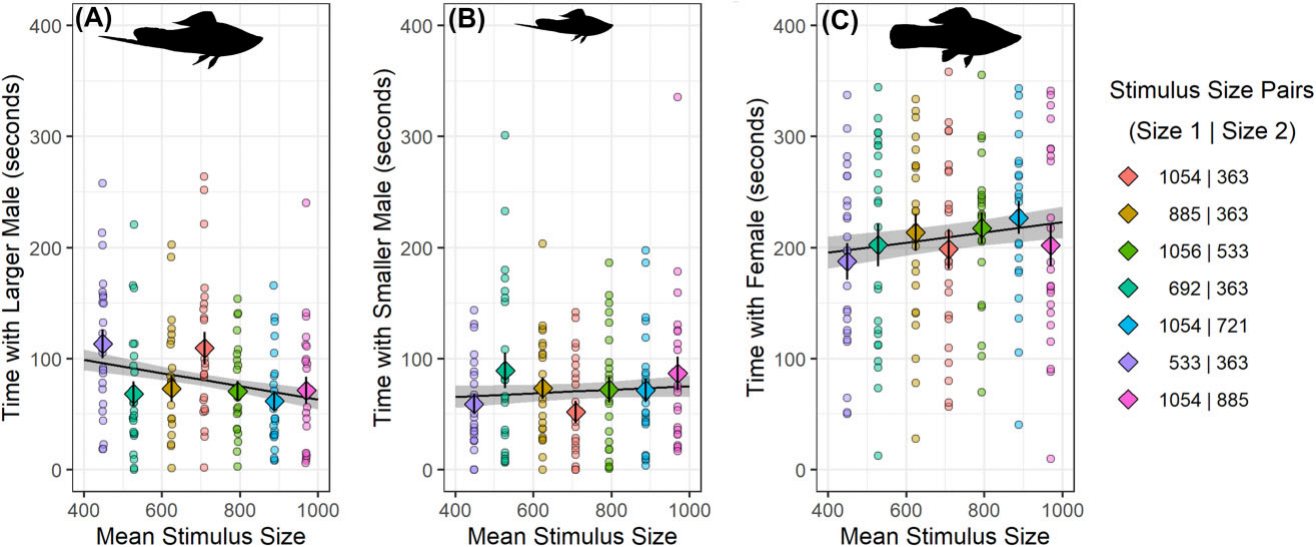
Focal males assessed the mean size of stimulus males, and adjusted their time spent with the defended female accordingly. As the mean size of stimulus males (*x*-axis) increased, focal males spent (A) less time (*y*-axis) with the larger stimulus male, (B) a similar amount of time with the smaller stimulus male, and (C) more time with the female. Solid black regression lines and error ribbons show estimate and standard errors from statistical models as described in Main Text. Colored circles indicate raw data and are color-coded to stimulus pairs. Diamonds with error bars represent mean values and standard errors for each stimulus size pair; the sizes (lateral area, mm^2^) of the stimuli in each pair are shown in the legend.

The best fit model predicting the amount of time the focal male spent with the larger stimulus male included only the mean size of the two stimulus males (the “social mean” model, Table 1). Using the ratio of model weights, this model had 3.17 times more empirical support than the next best-fit model. The second-place model included the mean size of both stimulus males along with the proportional difference in size between the focal and larger stimulus males; combined, the two best-fit models accounted for 75% of model weight (Table 1). Coefficient estimates from the social mean model showed that, as the mean size of both stimulus fish increased, the focal male spent less time with the larger stimulus fish (β ± SE = −10.44 ± 4.35; Fig. 4A). The third-place model included only an intercept term (i.e., was a “null” model) and was a better fit than the models using either self- or mutual-assessment. This suggests that commonly studied assessment strategies (mutual assessment and self-assessment) did not describe our data well. As described above, a similar ranking of models occurred if we assumed focal males attend to absolute, rather than proportional differences, in competitor size (Supplementary Table S1).

Given that males spent less time with the larger rival as mean rival size increased, we next asked what males did with their time if they were not spending it near the larger (i.e., most salient) competitor. A model of the time the focal male spent with the smaller stimulus male, as predicted by mean stimulus male size, found that focal males spent approximately the same amount of time with the smaller stimulus male as mean stimulus size increased (β ± SE = 2.87 ± 3.93, Fig. 4B). By contrast, a model predicting the time the focal male spent with the female, as predicted by mean stimulus size, found that focal males spent more time with the female as mean stimulus size increased (β ± SE = 8.02 ± 5.01, Fig. 4C).

The prior analysis built models of how the focal male allocated his time to the smaller stimulus male and the female, as predicted by the mean size of both males. However, it did not test how the fit of this model compared to models using other predictors (i.e., models in Table 1). Therefore, we conducted *post-hoc* model comparisons similar to that presented in Table 1, but with time spent with smaller male (instead of larger male) as the response variable. We found that the null model was the best-fit model (Supplementary Table S2). An additional model comparison using time spent with female as the outcome variable found that the social mean model was the best-fit model (Supplementary Table S3). In both model sets, other models were within 2AICc of the best-fit models, indicating lower confidence in the best-fit model, as compared to our analysis of time spent with the larger male (Table 1). However, in general, these results support the broad conclusions that focal males spent similar amounts of time with the smaller male, and more time with the female, as the size of the larger stimulus male increased.

## Discussion

Decision-making in contexts from mate choice to competition hinges on information-gathering, or assessment. Understanding how animals make decisions in fitness-relevant contexts, and therefore how behavior affects fitness, requires understanding how animals gather and use information. In animal contests, most literature suggests that fighting decisions are based on information about either an individual’s own fighting ability (RHP) or its own RHP relative to its opponent's [e.g., own/opponent (Enquist and Leimar 1983; Briffa et al. 2013)]. In some cases, this decision can be modified by information about other individuals. However, we have relatively little understanding of how animals gather information about multiple potential competitors, nor how they use this information to make fighting decisions (Sherratt and Mesterton-Gibbons 2013). Further, most research in contests assumes that competitors assess the absolute or ratio size differences of RHP linked traits like body size, despite the fact that proportional processing of magnitude-based traits is known to operate across taxa and modalities (Akre and Johnsen 2014). We found that male green swordtail fish decided how to spend their time in a contest based mainly on information about the social environment. Specifically, and somewhat surprisingly, the best-fit model suggested that neither proportional processing nor focal male RHP factored into focal male decisions; rather, males were most likely to make decisions based on the mean RHP of the two stimulus males. Below, we discuss the implications of our findings to assessment strategy research, and the relevance of incorporating alternative assessment strategies into these studies.

While there are relatively few studies on how animals assess during contests with multiple potential competitors, some work in this field has suggested that assessment of two potential opponents can influence fighting decisions. For example, male fallow deer *Dama dama* are more likely to intervene in an ongoing contest between two other males when those two rivals are of lower RHP than the intervening male; that is, males intervene more when they have greater ability than the two fighters (Jennings et al. 2021). Though our study was not focused on intervention behavior, our results are somewhat similar to this study: we found that focal males altered their fighting behavior based on the mean RHP of two potential competitors. Interestingly, focal males appeared not to base their fighting decisions on any information about their own fighting ability; instead, only information about the two stimulus males was included in the best-fit model [similar to “opponent-only” assessment in dyadic contests (Chapin et al. 2019)]. Theoretical models of third-party fighting behavior (specifically, related to eavesdropping on two competitors) suggest that individuals make decisions based on information about both their own RHP and the RHP (or fighting success) of potential opponents (Sherratt and Mesterton-Gibbons 2013). We note that the second best-fit model in our model set did include information about the focal male’s RHP (mutual assessment + social mean assessment); however, the social mean model was over three times more likely to describe the data (Table 1). One way to better connect our results to existing theory and experiments would be to test how male swordtails engage in intervention behavior when viewing other fighting individuals. If swordtails also base their fighting intervention decisions on purely social information (i.e., mean size of the opponents), it might suggest conserved behavioral tactics across varying contest scenarios.

When male swordtails decreased the time that they spent with the larger of two opponents, they mainly shifted their efforts to spending time near the female. That is, they focused more on resource defense. This makes logical sense if contest decisions are based on the mean size, or RHP, of both opponents. As the RHP of the nearby population of opponents increases, the potential for those opponents to gain access to the female might also increase; therefore, focal males could benefit from spending more time near the female in resource defense. We also found that focal males spent more time with the female than with either stimulus male (Fig. 4), suggesting that males were highly motivated to defend the resource.

While using video playback facilitated direct tests of the effect of proportional processing in contest assessment, there are shortcomings to our experimental approach that, if addressed, could especially improve conclusions related to how animals navigate social environments during contests. For instance, to facilitate tracking of the focal male and standardize engagement with the female across trials, we prevented the female from leaving a cylinder during trials. If she were allowed to move and make her own decisions, e.g., to perhaps select either the focal male or one of the stimulus males to associate with, this might affect focal male competitive behavior. Similarly, instead of using simulated males, if we had introduced two live opponent males, it would have allowed for a more direct test of how the social environment affects competitive decision-making. Finally, we only measured the time the focal male spent in given regions of the tank. Swordtails also produce several visual signals during mate choice and competitive interactions (Royle et al. 2005); by quantifying these, we could understand more about competitive decision-making. Given advances in the ability to track multiple unique individuals (Pereira et al. 2022) and automatically estimate poses like displays (Nath et al. 2019), these types of analyses are now more feasible than ever. However, we note that using video playback is the best way to tightly control relative opponent size and, therefore, test questions related to proportional processing. Given our findings that proportional processing may not play as strong of a role as expected in contest assessment in this system, future work may be able to sacrifice strict control over opponent size in favor of focusing on increasing aspects of social realism.

Our video playback approach had a clear benefit of allowing us to incorporate alternative mechanisms of size assessment into the study of animal contests. In particular, we were able to test whether males use proportional processing of size differences, or assessment of mean opponent size, when making fighting decisions. Our finding that males assessed the mean size of both opponents—especially that this model was much stronger than a model of either self-assessment or mutual assessment—suggests that tests of alternative size assessment strategies should be included in further theory and experiment in animal contests. The original formulation of one of the most common models of mutual assessment [the Sequential Assessment Model (SAM)] used the ratio of competitor RHP (own RHP/opponent RHP) as a metric of relative fighting ability (Enquist and Leimar 1983). However, as we describe, proportional processing invokes a different perceptual mechanism for relative RHP assessment, i.e., does not use a simple ratio. Further work may consider incorporating proportional processing into theoretical models (e.g., into the SAM), as well as into experimental tests of assessment strategies.

Overall, our results suggest that expanding beyond traditional models of contest assessment strategies—here, specifically, including proportional processing and the potential for social assessment—can better describe decisions in complex, yet realistic, competitive scenarios. Future studies could continue to work towards bringing ecological and perceptual realism into studies of assessment.

## Author contributions

All authors conceived of the research idea; E.M.C. and L.A.K. conducted the research; P.A.G. and E.M.C. analyzed the data and wrote the manuscript; all authors edited and approve the manuscript.

## Supplementary Material

icaf084_Supplemental_File

## Data Availability

All data and code used in this project are on the Brown Digital Repository and can be accessed at: https://doi.org/10.26300/yvht-6t15.
