# Supplementary material for: The Effects of Proportional Processing and Multiple Opponents on Contest Assessment in Male Green Swordtail Fish (Xiphophorus hellerii)
Source: Integr Comp Biol. 2025 Sep 16;65(4):822–31. doi: 10.1093/icb/icaf084 (PMC12530184; doi:10.1093/icb/icaf084)
Supplement: icaf084_Supplemental_File [file icaf084_supplemental_file.docx]

# **The effects of proportional processing and multiple opponents on contest assessment in male green swordtail fish (*Xiphophorus hellerii*)**

P.A. Green, L.A. Kelley, E.M. Caves

## **Supplement**

**Table S1.** Summary of model comparison results for time spent with the largest stimulus male, when using the absolute difference between stimulus males, or between the stimulus and focal male (i.e., when assuming absolute processing).

| **Model name** | **Terms** | **AICc** | **ΔAICc** | **weight** |
| --- | --- | --- | --- | --- |
| Social mean | Mean size of two stimulus males | 1813.8 | 0.00 | 0.51 |
| Mutual assessment + social absolute | Absolute difference with largest stimulus male | 1816.5 | 2.73 | 0.13 |
|  | Absolute difference of stimulus males |  |  |  |
| Mutual assessment + social mean | Absolute difference with largest stimulus male | 1816.5 | 2.75 | 0.13 |
|  | Mean size of two stimulus males |  |  |  |
| Null | N/A | 1817.3 | 3.52 | 0.09 |
| Mutual assessment | Absolute difference with largest stimulus male | 1817.8 | 3.99 | 0.07 |
| Social absolute | Absolute difference of stimulus males | 1819.2 | 5.36 | 0.04 |
| Self-assessment | Focal male size | 1819.4 | 5.61 | 0.03 |

**Table S2.** Summary of model comparison results for time spent with the smaller stimulus male. All models included a random effect of fish ID to account for repeated observations.

| **Model name** | **Terms** | **AICc** | **ΔAICc** | **weight** |
| --- | --- | --- | --- | --- |
| Null | N/A | 1796.2 | 0.00 | 0.298 |
| Social proportional | Proportional difference of stimulus males | 1796.4 | 0.25 | 0.263 |
| Social mean | Mean size of two stimulus males | 1797.8 | 1.57 | 0.136 |
| Mutual assessment | Proportional difference with largest stimulus male | 1798.2 | 2.00 | 0.110 |
| Self-assessment | Focal male size | 1798.3 | 2.10 | 0.104 |
| Mutual assessment + social proportional | Proportional difference with largest stimulus male | 1799.3 | 3.13 | 0.062 |
|  | Proportional difference of stimulus males |  |  |  |
| Mutual assessment + social mean | Proportional difference with largest stimulus male | 1801.0 | 4.80 | 0.027 |
|  | Mean size of two stimulus males |  |  |  |

**Table S3.** Summary of model comparison results for time spent with the female. All models included a random effect of fish ID to account for repeated observations.

| **Model name** | **Terms** | **AICc** | **ΔAICc** | **weight** |
| --- | --- | --- | --- | --- |
| Social mean | Mean size of two stimulus males | 1889.6 | 0.00 | 0.298 |
| Null | N/A | 1890.1 | 0.45 | 0.238 |
| Mutual assessment + social mean | Proportional difference with largest stimulus male | 1890.6 | 1.01 | 0.180 |
|  | Mean size of two stimulus males |  |  |  |
| Mutual assessment | Proportional difference with largest stimulus male | 1891.7 | 2.07 | 0.105 |
| Social proportional | Proportional difference of stimulus males | 1892.2 | 2.54 | 0.084 |
| Self-assessment | Focal male size | 1892.2 | 2.55 | 0.083 |
| Mutual assessment + social proportional | Proportional difference with largest stimulus male | 1896.0 | 6.32 | 0.013 |
|  | Proportional difference of stimulus males |  |  |  |
